# A framework for monitoring of new drugs in Sweden

**DOI:** 10.1080/03009734.2018.1550454

**Published:** 2019-01-28

**Authors:** Thomas Cars, Lars Lindhagen, Johan Sundström

**Affiliations:** a Department of Medical Sciences, Uppsala University, Uppsala, Sweden;; b Uppsala Clinical Research Center Uppsala University, Uppsala, Sweden

**Keywords:** Comparative effectiveness research, pharmacoepidemiology, propensity score, real-world evidence, sequential monitoring

## Abstract

In order to monitor the net public health benefit of new drugs, especially in the light of recent stepwise approval approaches, there is a need to optimize real-time post-marketing evaluation of new drugs using data collected in routine care. Sweden, with its unique possibilities for observational research, can provide these data. We herein propose a framework for continuous monitoring of the effectiveness, safety, and cost-effectiveness of new drugs, using prospectively determined protocols designed in collaboration between all relevant stakeholders. We believe that this framework can be a useful tool for healthcare authorities and reimbursement agencies in the introduction of new drugs.

## Introduction

Before the marketing of a new drug, its efficacy and safety are evaluated using randomized controlled trials (RCTs). RCTs are invaluable for establishing the relative treatment benefit of a new drug. However, the population eventually treated with a new drug often differs in many respects from the sample studied in the RCTs. In routine care, drugs are often used by elderly patients with more comorbidities and with multiple concomitant drug use. RCTs are also typically powered to detect only the most common adverse events. Consequently, rare or late adverse effects are not expected to be identified in RCTs. Hence, understanding of the realizable net public health benefit from a new drug requires other study designs than the RCT. Some of the data needed for such estimations may be available before the new drug is marketed, but other data are not available until the drug is actually used. Society needs these estimations as soon as possible, for determination of long-term safety, net public health benefit, and regulatory decisions. We herein propose a framework for such studies, using data collected in routine care.

## Observational studies of comparative effectiveness and safety

Comparative effectiveness research (CER) involves the comparison of healthcare interventions, aiming to produce evidence regarding the effectiveness and safety of medical products (1). A drug’s efficacy is defined as ‘the extent to which a specific health intervention produces a beneficial result under ideal conditions’ (i.e. a response to the question: ‘Can it work?’), whereas effectiveness can be defined as ‘the extent to which a specific health intervention produced a beneficial result when deployed in the field under routine conditions’ (i.e. a response to the question: ‘Does it work in practice?’) (2). The ultimate goal of comparative effectiveness research is to improve health by developing and disseminating evidence to patients, healthcare professionals, and policy-makers regarding the effectiveness of specific interventions. The exponential developments in the quantity, quality, and availability of digital healthcare data generated in routine care hold great promise for development of observational CER in digitally mature countries. This development parallels a trend towards more use of adaptive licensing of new drugs (3), with healthcare authorities and regulators relying increasingly on observational data for evaluation of drug effectiveness and safety (4–6).

## Unique possibilities in Sweden

Sweden provides unique opportunities for observational CER. The advantages lie in the country’s civic registration system involving a 12-digit personal identity number, unique to all Swedish citizens (7), and the fact that all residents have universal access to healthcare with a negligible co-payment for healthcare visits, hospitalizations, and drugs (8). Using the personal identity number to link healthcare data to a variety of nationwide health registers (classifying diagnoses using the International Classification of Diseases [ICD] system (9), surgical procedures using the Nordic Medico-Statistical Committee Classification of Surgical Procedures [NCSP] system (10), and filled drug prescriptions using the Anatomical Therapeutic Chemical Classification [ATC] system (11)), quality registers, and registers on sociodemographics and socioeconomics allows for research on large populations with near-zero loss to follow-up. Although many countries have registry data on prescription drugs at the individual level in ambulatory care, such data are often missing for drugs administered in hospitals (12, 13). Lack of individual-level data for hospital-based drugs is a large and increasing problem, since we are currently observing a trend towards more hospital-administered drugs.

Electronic health records (EHRs) provide an opportunity to include hospital drugs in observational CER. Sweden started to implement EHRs in the 1990s, and all regions had implemented EHRs in all healthcare areas by 2012 (14). Today, eight different EHR systems account for 97% of all EHR usage in Sweden, and most regions in Sweden have chosen to implement one single EHR system in their region (15). EHRs also contain more detailed clinical information than data from national health registries. Since the medical records are recorded as part of patient care, EHRs are instantly updated. This opens up for new and better opportunities to monitor treatments continuously with regard to its utility, safety, and cost-effectiveness.

In a PhD thesis, ‘Real-Time Monitoring of Healthcare Interventions in Routine Care. Effectiveness and Safety of Newly Introduced Medicines’ (16), we developed and validated a ‘sequentially evaluated non-randomized comparative effectiveness (SENCE)’ framework for continuous follow-up of new treatments using data from routine care in Sweden. This model is based on EHR data but can also utilize data from other sources. The model is built to collect and analyze data continuously as soon as new information is generated in the clinical data sources, making it possible to evaluate drugs sequentially and provide observational effectiveness evidence in as timely a manner as possible. The following is a summary of the proposed framework.

### Proposal for a framework for continuous post-marketing monitoring of new drugs in Sweden

We have developed a generic sequential cohort model for real-time head-to-head (drug A versus drug B) comparisons of new drugs when used in routine care. We propose that this model is set up prospectively before a new drug enters the market, and sequential monitoring is launched when the drug is marketed. Other interventions than drugs may also be analysed. To maximize the credibility and utility of the results produced by the model, we propose that this framework is carried out in collaboration between all relevant stakeholders (regulators, payers, and manufacturers).

## Process

Before the launch of a new therapy, regulators and authorities set the requirements for the post-marketing monitoring of the new drug. A project steering group and a scientific project group are formed. The steering group includes representatives from regulators, payers, and the drug manufacturer. The scientific project group includes experts in the actual therapeutical area, epidemiologists, and statisticians.

*Initiation phase*. In the initiation phase, a study protocol, statistical analysis plan (SAP), and an ethics committee application are developed by the project group. Permission to extract and include data is also obtained from data holders. A significant amount of work is dedicated to meticulously defining samples, data sources, exposures, best practice comparator drugs, covariates, and outcomes. A central task in this phase is the development of the causal models (17) and mimicking a target clinical trial (18). For development of causal models, we propose using the directed acyclic graphs (DAGs) approach (www.dagitty.net) in order to minimize potential bias (19). Statistical models used in sequential cohort design are defined (20–22). Before entering the data extraction and data management phase, all assumptions and the protocol are agreed upon between all stakeholders in the steering group, documented, and made available in the public domain (23).

The monitoring should begin when the drug is marketed. The *data extraction and data management phase* and the *data analysis phase* (see below) are repeated until stable results have been obtained.

*Data extraction and data management phase*. In this phase, data are extracted from EHRs and pseudonymized. Data from EHRs may also be linked to other data sources if necessary and defined in the initiation phase. All data should undergo quality checks including but not limited to logical checks, outlier detection, and investigation of missingness patterns. This step results in an analysis database and a data management report.

**Figure 1.**
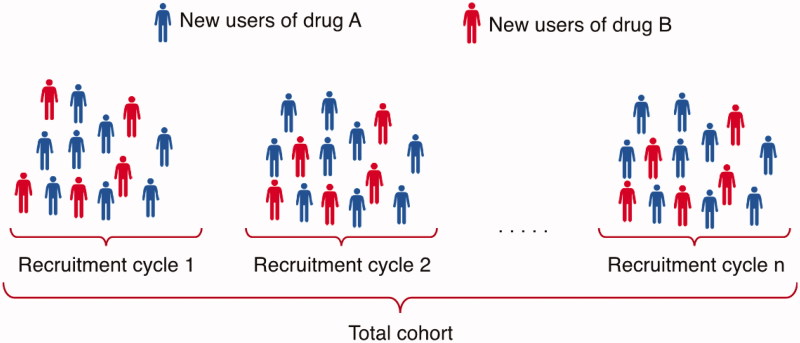
In each recruitment cycle, new users of drug A and new users of drug B are included and added to the cohort to continuously increase the study sample size.

*Data analysis phase*. In the analysis phase, data are analysed according to the SAP. The model is sequentially updated in order to evaluate data as they are collected, at a frequency determined by the projected uptake of the drug on the market. In a proof-of-concept study, we updated the model every six months, but the model can be updated at any desired frequency. In each time period (recruitment cycle), new users of the new treatment (drug A) and new users of a comparator treatment (drug B) are included and added to the cohort to continuously increase the study sample size (Figure 1).

**Figure 2.**
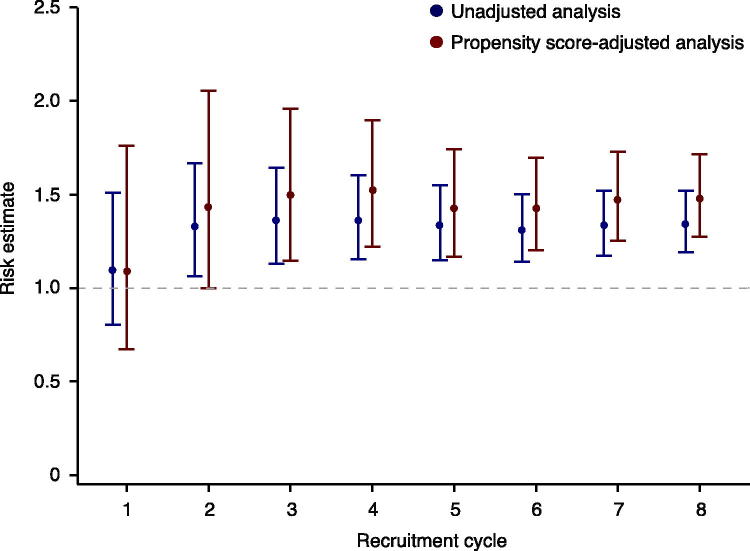
Effect estimates are published after each recruitment cycle.

At the end of each recruitment cycle, a propensity score (the probability of treatment assignment conditional on observed baseline covariates) (24) is estimated for all patients, and all patients are assessed for study outcomes. At the end of each recruitment cycle, a comparative effectiveness and/or comparative safety analysis is carried out for the entire sample (all new users of drugs A and B over the whole time at risk) and plotted sequentially for each recruitment cycle (Figure 2). Results are continuously made available in a timely fashion in the public domain. In this observational monitoring framework, we have chosen not to account for the sequential nature of the analysis. The rationale for this is that the intention of the sequential analysis was never to point out a single significant estimate or to terminate the study when a satisfactory result was observed. As a result of this strategy, each confidence interval presented in Figure 2 is only valid one at a time.

**Figure 3.**
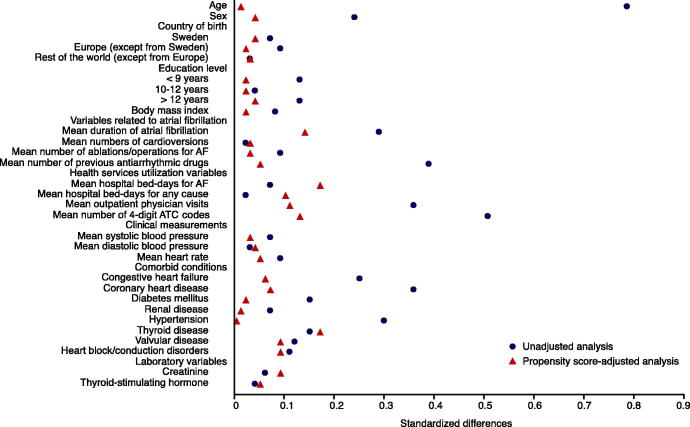
Standardized differences in baseline covariates between new users of drug A and new users of drug B before and after adjusting on the propensity score. A standardized difference <0.1 indicates negligible imbalance.

The goal of using a propensity score is to achieve covariate balance between groups of treated and controls, which is a fundamental step in the proposed process. Several methods to assess balance have been proposed. One frequently used method is to estimate standardized differences and represent the number of standard deviations by which the two groups differ (25). We propose to visualize the standardized differences using the plot presented in Figure 3.

**Figure 4.**
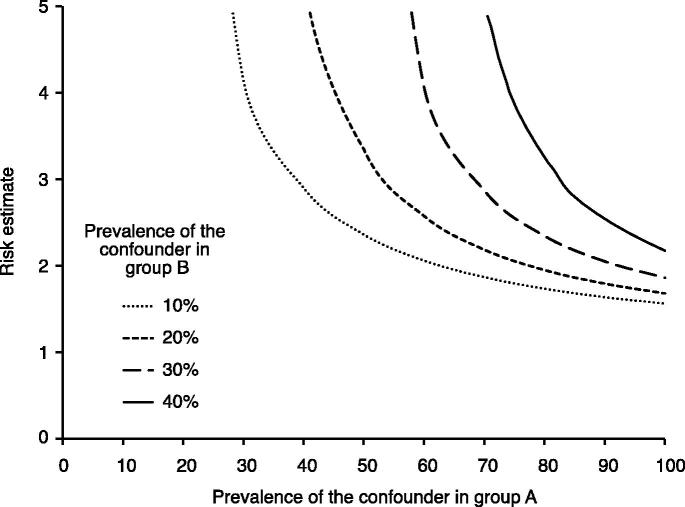
Evaluation of how powerful an unmeasured confounder would have to be to change the observed results. For example, if the prevalence of a potential unmeasured confounder is 40% in the drug A group (*x*-axis) and 10% in the drug B group, then the unmeasured confounder must have a risk estimate (hazard ratio) of the outcome close to 3 to fully explain the advantage of drug A over drug B.

The legitimacy of causal inference in observational studies is based on the assumption that no unmeasured confounding exists. This is a very strong assumption, and analyses should therefore be accompanied by sensitivity analyses investigating how the study findings may be affected by the presence of unmeasured confounding. We have in this framework included one commonly used approach proposed by Lin et al. (26) that involves evaluating how powerful an unmeasured confounder would have to be to change the observed results (Figure 4).

## Conclusion

In order to ensure the effectiveness, safety, and cost-effectiveness of new drugs, especially in the light of recent stepwise approval approaches, there is a need for regulators, payers in healthcare, and the pharmaceutical industry to optimize real-time post-marketing evaluation of new drugs using data collected in routine care. Sweden, with its unique possibilities for observational research, can have a strong position in the post-marketing monitoring of new drugs. We suggest that the herein proposed framework can provide timely and comprehensive observational evidence of effectiveness, safety, and costs of new drugs, using a prospectively determined protocol designed in collaboration between all relevant stakeholders. We further suggest that analyses are repeated regularly as long as they provide value, and that all results are published without delay in the public domain. We believe that this framework can be a useful tool for healthcare authorities and reimbursement agencies in the introduction of new drugs.

## References

[1] Conway PH, Clancy C Comparative-effectiveness research—implications of the federal coordinating council’s report. N Engl J Med. 2009;361:328–30. 19567829 10.1056/NEJMp0905631

[2] Haynes B Can it work? Does it work? Is it worth it? The testing of healthcareinterventions is evolving. Bmj. 1999;319:652 10480802 10.1136/bmj.319.7211.652 PMC1116525

[3] Eichler HG, Baird LG, Barker R, Bloechl-Daum B, Børlum-Kristensen F, Brown J, et al. From adaptive licensing to adaptive pathways: delivering a flexible life-span approach to bring new drugs to patients. Clin Pharmacol Ther. 2015;97:234–46. 25669457 10.1002/cpt.59 PMC6706805

[4] Sherman RE, Anderson SA, Dal Pan GJ, Gray GW, Gross T, Hunter NL, et al. Real-world Evidence - What Is It and What Can It Tell Us? N Engl J Med. 2016;375:2293–7. 27959688 10.1056/NEJMsb1609216

[5] European Medicines Agency (EMA). Annual Report 2017 [Internet]. [cited 2018 Jun 1]; Available from: http://www.ema.europa.eu/docs/en_GB/document_library/Annual_report/2018/04/WC500248201.pdf

[6] Tandvårds- och läkemedelsförmånsverket (TLV). Mesta möjliga hälsa för skattepengarna [Internet]. [cited 2018 Jun 1] Available from: https://www.tlv.se/om-oss/press/nyheter/arkiv/2018-02-22-mesta-mojliga-halsa-for-skattepengarna.html

[7] Ludvigsson JF, Otterblad-Olausson P, Pettersson BU, Ekbom A The Swedish personal identity number: possibilities and pitfalls in healthcare and medical research. Eur J Epidemiol. 2009;24:659–67. 19504049 10.1007/s10654-009-9350-y PMC2773709

[8] Anell A The public-private pendulum-patient choice and equity in Sweden. N Engl J Med. 2015;372:1–4. 25551523 10.1056/NEJMp1411430

[9] WHO Classifications [Internet. ] [cited 2017 ;Sep 14]; Available from: http://www.who.int/classifications/icd/en/

[10] NOMESCO NOMESCO Classification of Surgical Procedures [Internet. ] 2010 [Cited 2017 ;Sep 14]; Available from: https://norden.diva-portal.org/smash/get/diva2:970547/FULLTEXT01.pdf

[11] WHO Collaborating Centre for Drug Statistics Methodology ATC/DDD Index 2017 [Internet]. [Cited 2018 ;Feb 5]; Available from: https://www.whocc.no/

[12] Ferrer P, Ballarín E, Sabaté M, Laporte J-R, Schoonen M, Rottenkolber M, et al. Sources of European drug consumption data at a country level. Int J Public Health. 2014;59:877–87. 24875352 10.1007/s00038-014-0564-8

[13] Larsen MD, Cars T, Hallas J A minireview of the use of hospital-based databases in observational inpatient studies of drugs. Basic Clin Pharmacol Toxicol. 2013;112:13–8. 22901097 10.1111/j.1742-7843.2012.00928.x

[14] Jerlvall L, Pehrson T eHälsa i landstingen (In Swedish) [Internet. ]. 2012 [Cited 2018 ;Jan 15]; Available from: https://www.inera.se/globalassets/om-inera/styrdokument-och-rapporter/ehalsa-i-landstingen/ehlsa_i_landstingen_slit_2012.pdf

[15] Jerlvall L, Pehrsson T eHälsa och IT i landstingen [Internet. ]. [cited 2018 ;Jun 1]; Available from: https://www.inera.se/globalassets/om-inera/styrdokument-och-rapporter/ehalsa-i-landstingen/ehalsa-it-ilandstingen-2017.pdf

[16] Cars T Real-time monitoring of healthcare interventions in routine care: effectiveness and safety of newly introduced medicines [Internet. ]. 2016 [Cited 2018 ;Jan 15]; Available from: http://uu.diva-portal.org/smash/get/diva2:1015130/FULLTEXT01.pdf

[17] Hernán MA, Robins JM Causal inference book [Internet]. Boca Raton: Chapman & Hall/CRC, forthcoming, 2018 Available from. https://www.hsph.harvard.edu/miguel-hernan/causal-inference-book/

[18] Hernán MA, Robins JM Using big data to emulate a target trial when a randomized trial is not available. Am J Epidemiol. 2016;183:758–64. 26994063 10.1093/aje/kwv254 PMC4832051

[19] Textor J, Hardt J, Knuppel S DAGitty: a graphical tool for analyzing causal diagrams. Epidemiology. 2011;22:745 10.1097/EDE.0b013e318225c2be 21811114

[20] Schneeweiss S, Gagne JJ, Glynn RJ, Ruhl M, Rassen JA Assessing the comparative effectiveness of newly marketed medications: methodological challenges and implications for drug development. Clin Pharmacol Ther. 2011;90:777–90. 22048230 10.1038/clpt.2011.235

[21] Stürmer T, Wyss R, Glynn RJ, Brookhart MA Propensity scores for confounder adjustment when assessing the effects of medical interventions using nonexperimental study designs. J Intern Med. 2014;275:570–80. 24520806 10.1111/joim.12197 PMC4037382

[22] Cars T, Lindhagen L, Malmström R, Neovius M, Schwieler J, Wettermark B, et al. Effectiveness of drugs in routine care: a model for sequential monitoring of new medicines using dronedarone as example. Clin Pharmacol Ther. 2018;103:493–501. 28560722 10.1002/cpt.751

[23] ENCePP E-Register of Studies [Internet. ]. [cited 2018 ;Jun 1]; Available from: http://www.encepp.eu/encepp_studies/e_register.html

[24] Rosenbaum PR, Rubin DB The central role of the propensity score in observational studies for causal effects. Biometrika 1983;70:41–55.

[25] Austin PC Goodness-of-fit diagnostics for the propensity score model when estimating treatment effects using covariate adjustment with the propensity score. Pharmacoepidem Drug Safe. 2008;17:1202–17. 10.1002/pds.1673 18972454

[26] Lin DY, Psaty BM, Kronmal RA Assessing the sensitivity of regression results to unmeasured confounders in observational studies. Biometrics 1998;54:948–63. 9750244

